# Renal Ischemia/Reperfusion Injury in Soluble Epoxide Hydrolase-Deficient Mice

**DOI:** 10.1371/journal.pone.0145645

**Published:** 2016-01-04

**Authors:** Ye Zhu, Maximilian Blum, Uwe Hoff, Tim Wesser, Mandy Fechner, Christina Westphal, Dennis Gürgen, Rusan Ali Catar, Aurelie Philippe, Kaiyin Wu, Gordana Bubalo, Michael Rothe, Steven M. Weldon, Duska Dragun, Wolf-Hagen Schunck

**Affiliations:** 1 Nephrology and Intensive Care Medicine, Campus Virchow and Center for Cardiovascular Research, Charité Medical Faculty, Berlin, Germany; 2 The fifth affiliated hospital of Sun Yat-sen University, Guangdong Province, Zhuhai, China; 3 Max Delbrueck Center for Molecular Medicine, Berlin, Germany; 4 Institute of Pathology, Charité Campus Mitte, 10117, Berlin, Germany; 5 Lipidomix GmbH, Berlin, Germany; 6 Boehringer Ingelheim Pharmaceuticals Inc., 900 Ridgebury Road, Ridgefield, United States of America; Institute of Neurology (Edinger-Institute), GERMANY

## Abstract

**Aim:**

20-hydroxyeicosatetraenoic acid (20-HETE) and epoxyeicosatrienoic acids (EETs) are cytochrome P450 (CYP)-dependent eicosanoids that play opposite roles in the regulation of vascular tone, inflammation, and apoptosis. 20-HETE aggravates, whereas EETs ameliorate ischemia/reperfusion (I/R)-induced organ damage. EETs are rapidly metabolized to dihydroxyeicosatrienoic acids (DHETs) by the soluble epoxide hydrolase (sEH). We hypothesized that sEH gene (EPHX2) deletion would increase endogenous EET levels and thereby protect against I/R-induced acute kidney injury (AKI).

**Methods:**

Kidney damage was evaluated in male wildtype (WT) and sEH-knockout (KO)-mice that underwent 22-min renal ischemia followed by two days of reperfusion. CYP-eicosanoids were analyzed by liquid chromatography tandem mass spectrometry.

**Results:**

Contrary to our initial hypothesis, renal function declined more severely in sEH-KO mice as indicated by higher serum creatinine and urea levels. The sEH-KO-mice also featured stronger tubular lesion scores, tubular apoptosis, and inflammatory cell infiltration. Plasma and renal EET/DHET-ratios were higher in sEH-KO than WT mice, thus confirming the expected metabolic consequences of sEH deficiency. However, CYP-eicosanoid profiling also revealed that renal, but not plasma and hepatic, 20-HETE levels were significantly increased in sEH-KO compared to WT mice. In line with this finding, renal expression of Cyp4a12a, the murine 20-HETE-generating CYP-enzyme, was up-regulated both at the mRNA and protein level, and Cyp4a12a immunostaining was more intense in the renal arterioles of sEH-KO compared with WT mice.

**Conclusion:**

These results indicate that the potential beneficial effects of reducing EET degradation were obliterated by a thus far unknown mechanism leading to kidney-specific up-regulation of 20-HETE formation in sEH-KO-mice.

## Introduction

Renal ischemia-reperfusion (I/R) is one of the major causes of acute kidney injury (AKI) [1]. Ischemic AKI greatly contributes to patient morbidity and mortality in various clinical settings such as cardiovascular surgery and renal transplantation [2–5]. Even after complete recovery, AKI is an independent risk factor for the development of chronic kidney disease [6,7]. An effective therapy of ischemic AKI is still lacking [8]. The complex pathophysiology of AKI involves hemodynamic alterations, inflammation, endothelial dysfunction, and tubular epithelial cell injury [1,8,9].

Recent preclinical studies indicate that arachidonic acid (AA) metabolites generated by cytochrome P450 (CYP) enzymes play an important role in the development of I/R-injury in the kidney [10–12], heart [13,14] and brain [15,16]. These metabolites include 20-hydroxyeicosatetraenoic acid (20-HETE), the primary product of CYP4A/CYP4F-catalyzed AA ω-hydroxylation, and epoxyeicosatrienoic acids (EETs) produced by AA epoxygenases of the CYP2C and CYP2J subfamilies [17–20]. Whereas inhibition of 20-HETE synthesis reduced I/R injury in the heart and brain, corresponding studies in the kidney yielded controversial results that seem to be related to the model systems used, bilateral [10] versus unilateral ischemia [11], as confirmed in a follow-up study [12]. 20-HETE is excessively released during renal ischemia [11] and may initiate I/R injury by promoting vasoconstriction [21] as well as endothelial dysfunction [22] and tubular epithelial cell apoptosis [23]. Conversely, 20-HETE mediated inhibition of tubular salt reabsorption is required for normal kidney function [24] and may play a protective role in renal I/R injury by reducing oxygen utilization in the reperfusion phase [10,12].

EETs share the capacity of 20-HETE to inhibit tubular sodium transport but show a profile of vascular activities that opposes that of 20-HETE [21,24]. EETs mediate vasodilator responses and have been identified as the major endothelium-derived hyperpolarizing factor in renal arterioles [21,25]. EETs repress pro-inflammatory activation of endothelial cells by inhibiting cytokine-induced nuclear factor-κB (NF-κB) activation and vascular cell adhesion molecule 1(VCAM-1) expression [26]. Moreover, EETs have the potential of inhibiting hypoxia/reoxygenation-induced apoptosis and cell death as first shown in cultured endothelial cells [27] and cardiomyocytes [28]. EETs are rapidly metabolized to less active dihydroxyeicosatrienoic acids (DHETs) by the action of the soluble epoxide hydrolase (sEH) [29]. The sEH enzyme is encoded by the EPHX2 gene and consists of an N-terminal phosphatase and C-terminal hydrolase domain. Inhibitors targeting the hydrolase domain increase the endogenous EET levels and have been shown to be antihypertensive and anti-inflammatory, and to protect the brain, heart and kidney from damage [30,31]. Renoprotective actions of sEH-inhibitors were demonstrated in various animal models of cardiovascular disease [32] and specifically also in mouse models of renal I/R-injury [33] and obstructive nephropathy [34]. Human studies revealed significant associations between genetic variation in EPHX2 and allograft function after kidney transplantation [35] as well as with the risk of IgA nephropathy progression [36].

In the present study, we used sEH-knockout and corresponding wildtype mice to test the hypothesis that sEH gene deficiency protects against renal I/R-injury. Unexpectedly, we observed that EPHX2 deletion aggravated the disease process. Searching for the potential mechanisms, we found that the sEH-KO mice displayed kidney-specific upregulation of 20-HETE formation.

## Methods

### Animals

The sEH-KO mice were originally established by Boehringer Ingelheim Pharmaceuticals, Inc [37] and were then further backcrossed for nine generations onto C57BL/6ByJ before being used in our studies [38]. sEH-KO mice and corresponding WT littermates were kept under specific pathogen free (SPF) conditions with a standard 12:12 h light-dark cycle and had ad libitum access to water and standard chow. This study was carried out in strict accordance with the recommendations in the Guide for the Care and Use of Laboratory Animals of the National Institutes of Health and the ethics policies of Charité-University Berlin and the Land Berlin. The protocol was approved by the Committee on the Ethics of Animal Experiments of the Charité and Land Berlin (Permit Number: G0121/11).

### Genotyping

Genomic DNA was extracted from mouse tail biopsies for polymerase chain reactions (PCR)-based genotyping. The primers (BioTez, Berlin-Buch GmbH) were as follows: SEPOH-FP, 5′-CCACCTACCTTGTGCTTGCC-3′, SEPOH-RP, 5′-GGGAAGAGGGGAAGGATTGT-3′, and LTR2, 5′-AAATGGCGTTACTTAAGCTAGCTTGC-3′. The PCR products were separated on 2% agarose gels and visualized under UV light after staining with GelRed^TM^ (41003, Biotium, Hayward, USA). The genotype-specific PCR products had a size of 308 (homozygous WT) and 230 bp (homozygous sEH-KO mice).

### Determination of sEH activities

Renal and hepatic cytosolic fractions were prepared as described previously [39]. The assay was performed at 37°C for 20 min in a final volume of 100 μL potassium phosphate buffer (0.1 M, pH 7.2) containing 50 μM 14,15-EET as substrate. The reactions were started by adding the cytosolic fraction (3.5 μg of protein) and terminated with 300 μl ethyl acetate. The remaining substrate and its product (14,15-DHET) were extracted and analyzed by reversed-phase high performance liquid chromatography (RP-HPLC) [39].

### Renal I/R injury

Male mice were used at the age of 10–13 weeks, 25–30 g in weight. Animals were anesthetized with isoflurane (“Forene”, Abbott GmbH & Co., KG Wiesbaden) and placed on a temperature-controlled heating table, maintaining the body temperature in the range of 36.5–37.5°C. After removal of the right kidney, ischemia was induced in the remaining kidney by applying a non-traumatic vascular clamp (FST, Essen, Germany) to the left renal pedicle for 22 min. Renal-reflow was confirmed after releasing the clamp by visual inspection. Before wound closure, 1 ml of pre-warmed (37°C) saline was placed in the abdominal cavity to prevent dehydration. For analgesia, mice received a single injection of buprenorphine followed by tramadol in drinking water during the next two days. Sham (control) groups went through the identical procedures including uninephrectomy, however, without clamping of renal pedicle. One day after surgery, the animals were individually placed in metabolic cages for urine collection over a period of 24 h. Blood, urine, and kidneys tissue samples were collected 48 h after I/R. The kidneys were cut in half through the long axis. One half of the kidney was fixed in 4% paraformaldehyde for paraffin embedding, while the second half was snap frozen in liquid nitrogen and stored at −80°C for subsequent mRNA, protein, or CYP-eicosanoid analysis. Before analysis, the stored samples were homogenized in liquid nitrogen using a Biopulverizer (BioSpec Products Inc., USA). Completely untreated sEH-KO and WT mice were used for evaluating the oxylipin profiles, sEH activities and Cyp4a12a expression under baseline conditions.

### Renal function and histology

Creatinine and urea nitrogen in serum and urine were measured by an automated chemistry analyzer. Histomorphologic analysis of Hematoxylin and Eosin (HE) and Periodic Acid-Schiff (PAS) stained renal paraffin embedded sections (2 μm) were used to determine acute tubular necrosis (ATN) score as described previously [40]. TUNEL staining was performed to detect DNA fragmentation associated with programmed renal cell death by In Situ Cell Death Detection Kit, TMR red (Roche Diagnostics GmbH, Mannheim, Germany) according to the manufacturer’s instructions. For the evaluation of monocyte/macrophage infiltration, acetone-fixed frozen renal sections (6 μm) were incubated with the mixture of primary antibodies rat-anti-mouse macrophage F4/80 (1:100, Serotec, Oxford, UK) and rat-anti-mouse CD11b (1:250, clone 1/70, Pharmingen, Oxford, UK) by immunofluorescence staining. The location of Cyp4a12a in renal sections was analyzed using an affinity purified antibody (1:200) raised in rabbit against a Cyp4a12a-specific peptide [39] without or after pre-saturation with the corresponding synthetic peptide. A goat anti-rabbit IgG Alex Red 568 conjugate (1:1000, Vector Labs, Burlingame, CA, USA) served as secondary antibody. Images were examined with a microscope and AxioVision digital imaging system (Zeiss, Jena, Germany) in 10 randomly chosen fields of view (FoV) at 200× or 400× magnification. The quantification of positive signals was evaluated as the percentage of total area per FoV.

### Quantitative analysis of mRNA expression

Total RNA was extracted with the Qiazol RNeasy Micro kit including DNase digestion (Qiagen, Hilden, Germany) and then reverse-transcribed into cDNA using a high-capacity cDNA reverse-transcription kit (Applied Biosystems, Foster City, CA, USA). Subsequent TaqMan analysis of Cyp4a12a and sEH mRNA expression was conducted as described previously [39]. The relative amount of gene transcript was calculated by using the standard curve method and then normalized on GAPDH.

### Western blot analysis

Aliquots (30 μg of protein per lane) of kidney or liver homogenates were separated by 10% SDS-PAGE and transferred onto PVDF membranes (GE Healthcare, Amersham, UK). Recombinant Cyp4a12a protein was included as positive control. The primary antibodies used were raised in rabbits against mouse Cyp4a12a [39], sEH (Cayman Chemicals, Ann Arbor, USA), and GAPDH (HyTest, Turku, Finland) and were applied in dilutions of 1:1000, 1:1000 and 1:20000, respectively. Anti-rabbit IgG peroxidase conjugate (1:10000, Jackson ImmunoResearch, West Grove, PA, USA) served as secondary antibody. Immunoreactive bands were detected by chemiluminescence using the Super Signal West Dura substrate (Thermo Scientific, Rockford, IL, USA) and quantified with the G:BOXChemi XL 1.4 imaging system (Syngene, Cambridge, UK).

### Plasma and tissue oxylipin profiles

Plasma and homogenized tissue (kidney and liver) samples were subjected to alkaline hydrolysis and solid-phase extraction was performed as described previously [41]. 10 ng of each 20-HETE-*d*6, 14,15-EET-*d*8, 14,15-DHET-*d*11, and 15-HETE-*d*8 (Cayman Chemicals, Ann Arbor, MI, USA) served as internal standards. Subsequent analysis of the endogenous eicosanoid profiles was performed by liquid chromatography tandem mass spectrometry (LC-MS/MS; Lipidomix GmbH, Berlin, Germany) as established previously [42]. Results are given in ng metabolites per ml plasma or per g of organ wet weight.

### Statistics

Statistical analysis was performed by using GraphPad Prism 5 software (GraphPad Inc., La Jolla, USA). All results were tested for normal distribution and expressed as mean ± standard error of mean (SEM). Two-tailed t-test was used for comparing the difference in terms of mean values between two different groups. The significance of variability among multi groups was evaluated by one-way ANOVA with a Bonferroni multiple comparison post-test. P<0.05 (*), <0.01 (**) and <0.001 (***) were considered as statistically significant.

## Results

### Confirmation of functional sEH gene disruption

**Fig 1:**
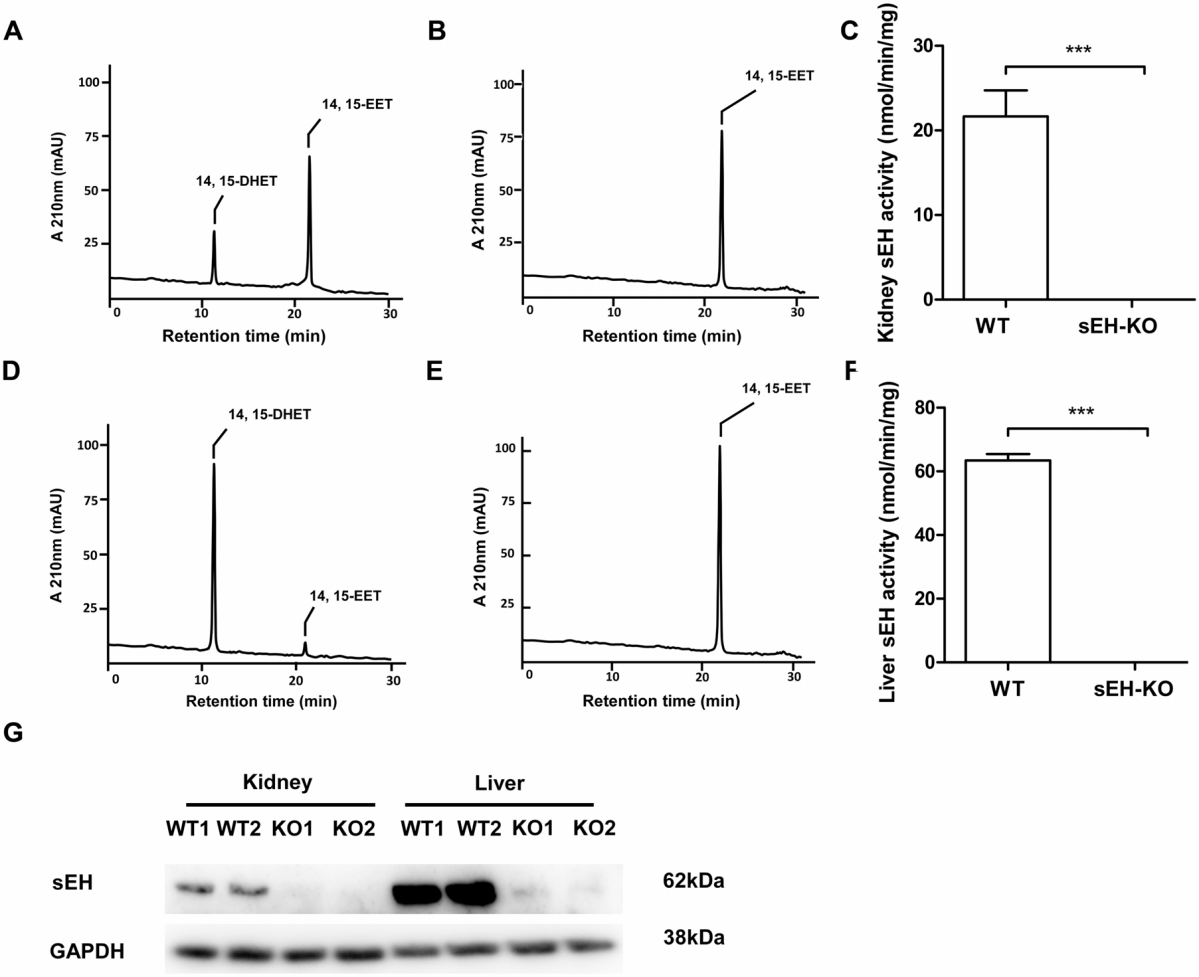
sEH gene disruption abolished sEH activities and sEH protein expression. Representative HPLC chromatograms showing the metabolism of 14,15-EET to 14,15-DHET by cytosolic fractions prepared from kidney and liver of WT (A and C) and sEH-KO (B and D) mice. The sEH activity in renal (C) and liver (F) cytosolic fractions from sEH-KO and WT mice revealed complete loss of activity by gene knock out. Data are given as mean ± SEM (n = 5–6 per group). Statistically significant differences were observed as indicated: ** p<0.01 vs WT. (G): Representative Western blot of liver and kidney homogenates from sEH-KO and WT mice.

Evaluation of renal and hepatic sEH activities was performed using 14,15-EET as natural substrate and analyzing its conversion to 14,15-DHET by RP-HPLC. The cytosolic fractions prepared from the organs of WT mice metabolized 14,15-EET with hydrolase activities of about 20 (kidney, Fig 1A–1C) and 60 nmol/min/mg (liver, Fig 1D–1F). In contrast, 14,15-EET hydrolysis was not catalyzed by any of the corresponding samples derived from homozygous sEH-KO mice (Fig 1A–1F). Polyclonal antibodies raised against recombinant mouse sEH recognized a 62 kDa protein band in the kidney and liver samples of WT but not sEH-KO mice (Fig 1G). Taken together, these results confirmed that sEH gene disruption resulted in a complete loss of functional sEH expression.

### I/R-induced impairment of renal function was aggravated in sEH-KO mice

**Fig 2:**
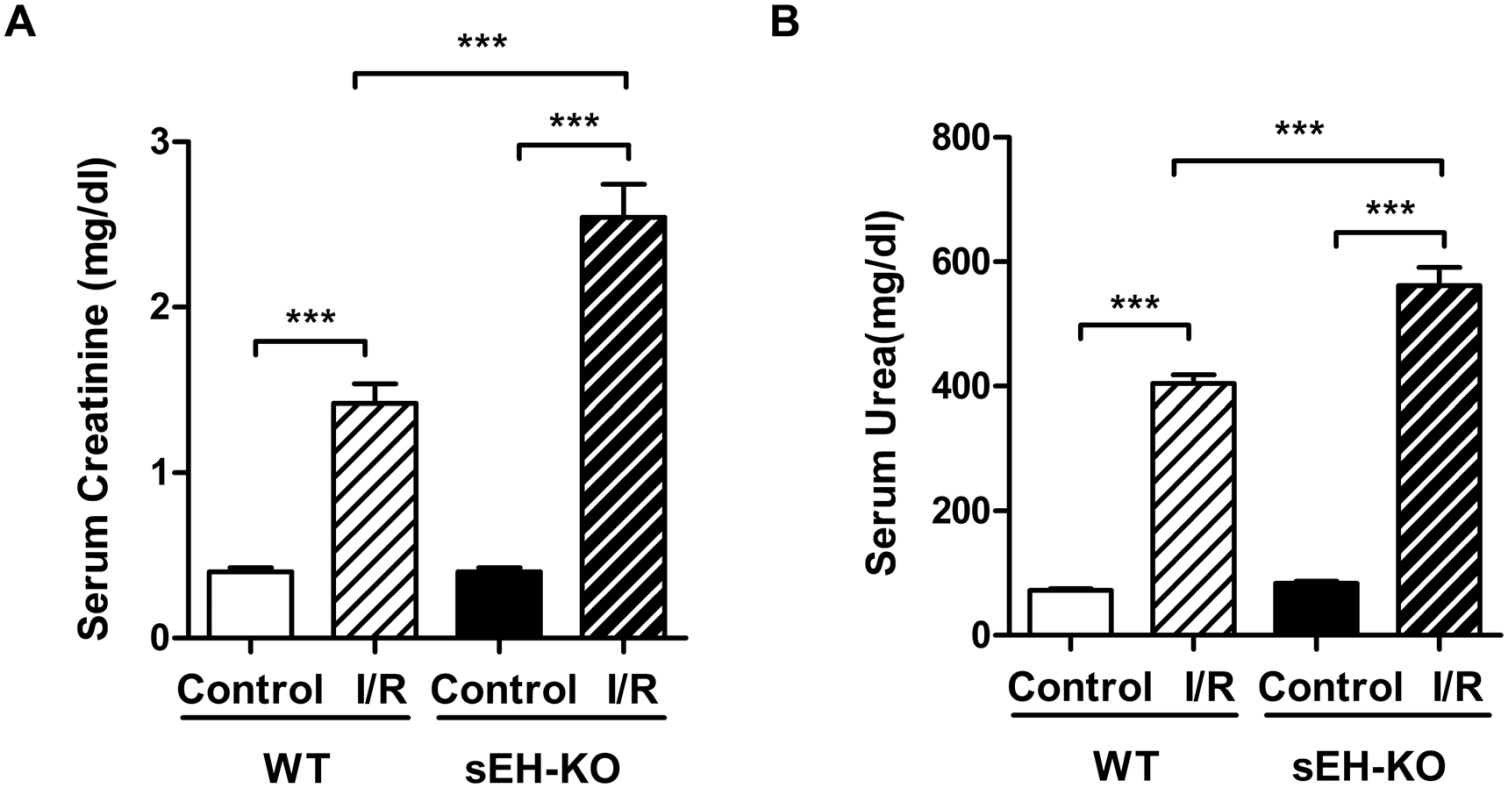
sEH gene disruption aggravated I/R-induced impairment of renal function. Kidney function was evaluated by measuring the serum levels of creatinine (A) and urea (B) two days after reperfusion. sEH-KO mice presented significantly stronger increases of serum creatinine and urea levels compared to the WT-I/R or uninephrectomized control groups. Data are given as mean ± SEM (n = 5–8 per group). ***p<0.001 vs WT.

Serum creatinine and urea levels were determined two days after reperfusion in order to evaluate the extent of I/R-induced impairment of renal function. In WT mice, I/R resulted in a 3.5-fold increase of serum creatinine (Fig 2A) and a 5.5-fold rise in serum urea (Fig 2B), compared with sham-operated uninephrectomized controls. The extent of I/R-induced impairment of renal function was more pronounced in sEH-KO than WT mice (creatinine: 2.54 ± 0.20 vs. 1.42 ± 0.12 mg/dl, *P*<0.001; urea: 561.6 ± 29.63 vs. 404.4 ± 13.93 mg/dl, *P*<0.001; Fig 2).

### sEH-KO mice displayed increased I/R-induced tubular damage and renal inflammation

**Fig 3:**
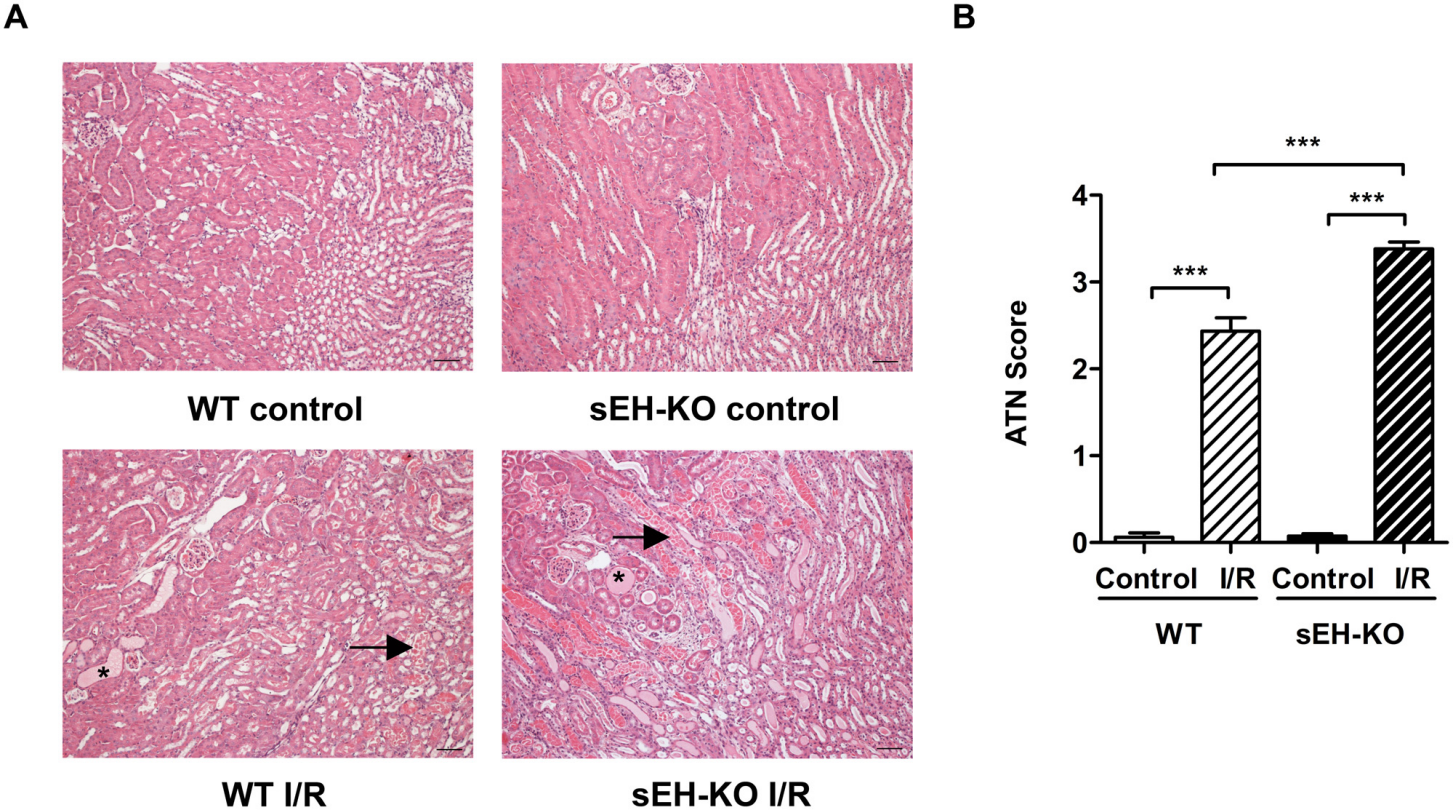
sEH gene disruption enhanced I/R-induced tubular damage. (A): Representative images of PAS-stained sections of kidneys harvested two days after reperfusion or sham surgery (magnification 200×, scale bar: 50 μm). Images were taken at the border of cortex and outer medulla. Arrows indicate necrotic tubules, and asterisks indicate tubular casts. (B): Evaluation of Acute Tubular Necrosis (ATN) score. sEH-KO mice subjected to I/R injury showed significantly increased tubular damage compared to the WT I/R or uninephrectomized control groups. Data are given as mean ± SEM (n = 5 per group). ***p<0.001 vs WT.

**Fig 4:**
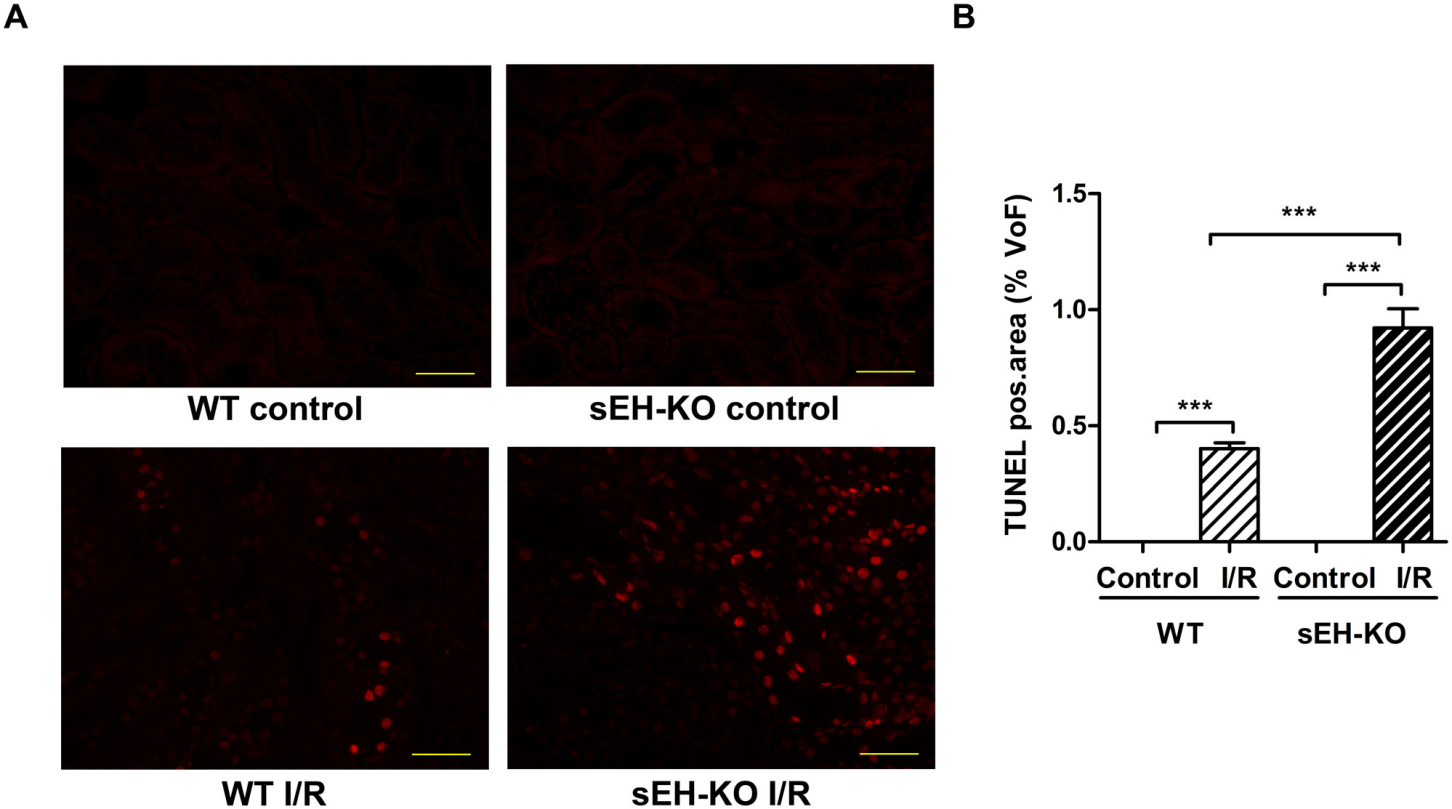
sEH gene disruption increased I/R-induced apoptosis of tubular epithelial cells. (A): Representative images of renal sections after TUNEL-staining (magnification 400×, Scale bar 100 μm). Apoptosis was detected in the kidneys of all mice subjected to I/R-injury but not in the corresponding control mice. (B): Quantification of apoptosis in the cortex and outer medulla of kidneys harvested two days after reperfusion. The intensity of positively stained nuclei was related to the area of each chosen field of view (FoV) in the renal sections. sEH-KO mice displayed significantly stronger apoptosis compared to the WT-I/R or control groups. Data are given as mean ± SEM (n = 5 per group). ***p<0.001 vs WT.

**Fig 5:**
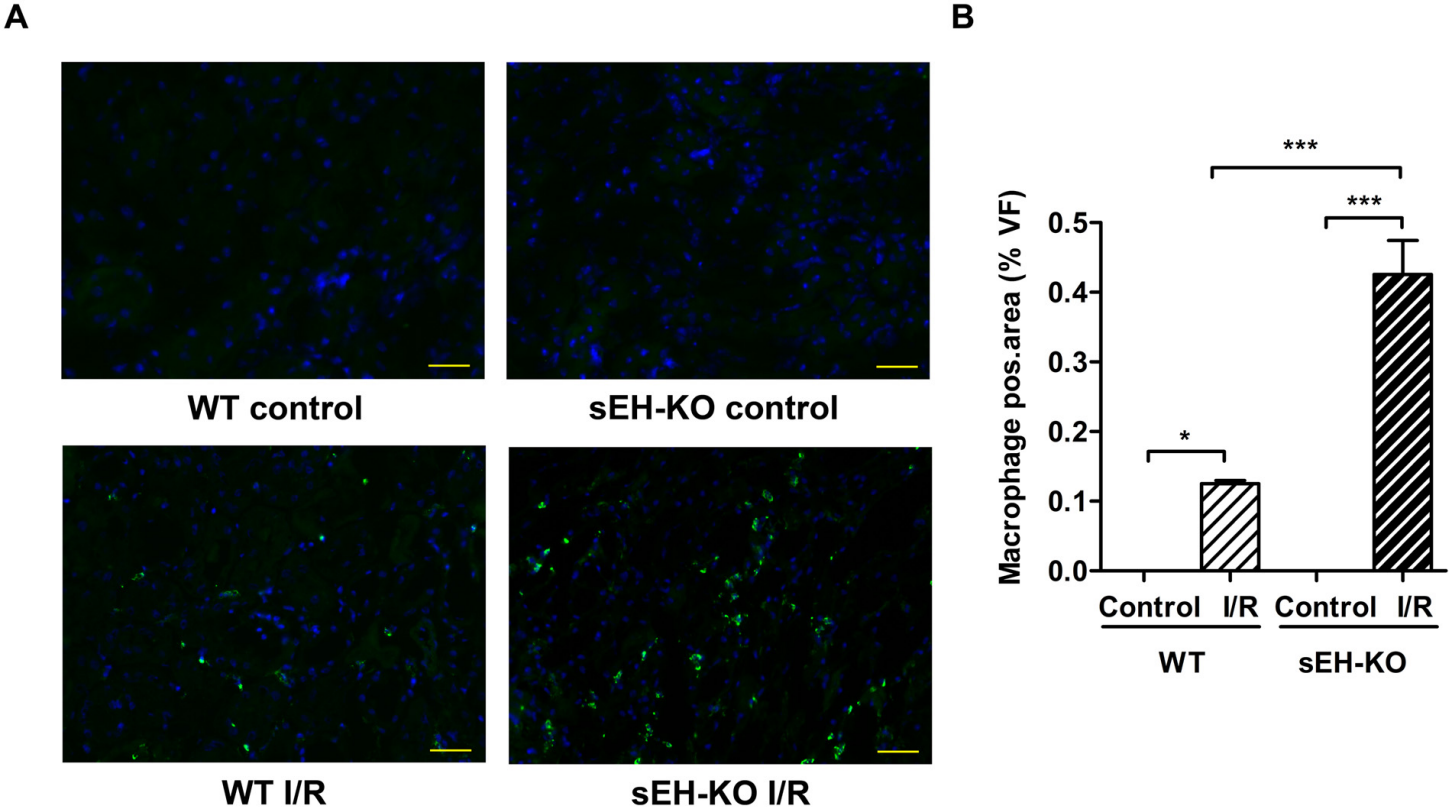
sEH gene disruption aggravated I/R-induced renal inflammation. (A): Representative images of renal sections stained for monocytes/macrophages to evaluate inflammatory cell infiltration as induced by renal I/R injury (magnification 400×, Scale bar 100 μm). (B): Quantification of inflammatory cell infiltration by evaluating the intensity ratio of positively stained inflammatory cells to the area of the high power view field. sEH-KO mice displayed significantly more inflammation compared to the WT-I/R or control groups. Data are given as mean ± SEM (n = 5 per group). * p<0.05, ***p<0.001 vs WT.

In line with the differences observed in renal functional impairment, sEH-KO mice showed higher tubular necrosis scores (Fig 3), stronger tubular apoptosis (Fig 4), and intensified inflammatory cell infiltration (Fig 5) compared with WT mice. The corresponding histological examinations were performed using the kidneys harvested two days after reperfusion. I/R-induced renal tubular damage was indicated by the occurrence of widened tubular lumina, exfoliated tubular epithelial cells, hyaline cast formation, and necrotic tubules (Fig 3A). Tubular damage was primarily detectable in the outer medulla and adjacent cortex following the vasculature along the collecting ducts. The degree of necrotic renal injury was significantly higher in sEH-KO than WT mice as quantified by the ATN score: 3.40 ± 0.09 vs. 2.50 ± 0.17, *P*<0.001; Fig 3B. The sEH-KO animals also showed augmented apoptosis of tubular epithelial cells as quantified by morphometric analysis of TUNEL staining (0.92 ± 0.08, vs 0.40 ± 0.02% per field of view (FoV), *P*<0.001; Fig 4A and 4B). I/R-induced inflammation was indicated by dense infiltration of monocytes/macrophages into the damaged zones of the outer medulla and renal cortex. Morphometric quantification revealed aggravated inflammatory cell infiltration in sEH-KO compared to WT mice (0.43 ± 0.049%, vs. 0.13 ± 0.004 per FoV, *P*<0.001; Fig 5A and 5B).

### Oxylipin analysis revealed increased renal 20-HETE formation in sEH-KO mice

**Fig 6:**
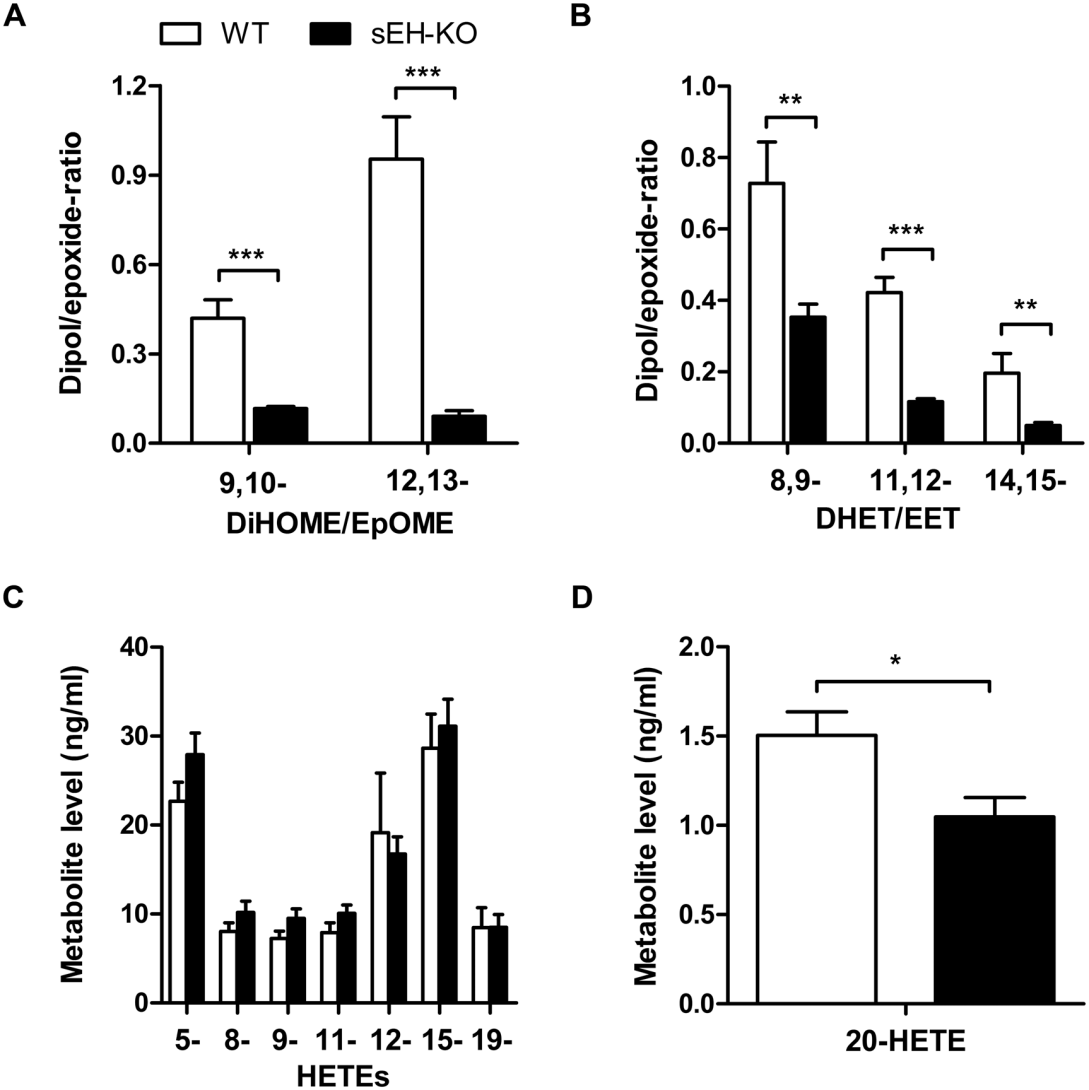
Plasma oxylipin profiles in WT and sEH-KO mice. (A): The conversion of linoleic acid-derived epoxides (EpOMEs) to the corresponding vicinal diols (DiHOMEs) was reduced in sEH-KO compared to WT mice as indicated by the decreased DiHOME/EpOME-ratios. (B): The conversion of AA-derived epoxides (EETs) to the corresponding vicinal diols (DHETs) was reduced in sEH-KO compared to WT mice as indicated by the decreased DHET/EET-ratios. (C): The plasma levels of AA-derived 5- through 19-HETE were not different in WT and sEH-KO mice. (D): Plasma 20-HETE levels were significantly lower in sEH-KO compared with WT mice. Results are given as mean ± SEM (n = 5–6 per group). Statistically significant differences were observed as indicated: * p<0.05, ** p<0.01, ***p<0.001 vs WT.

**Fig 7:**
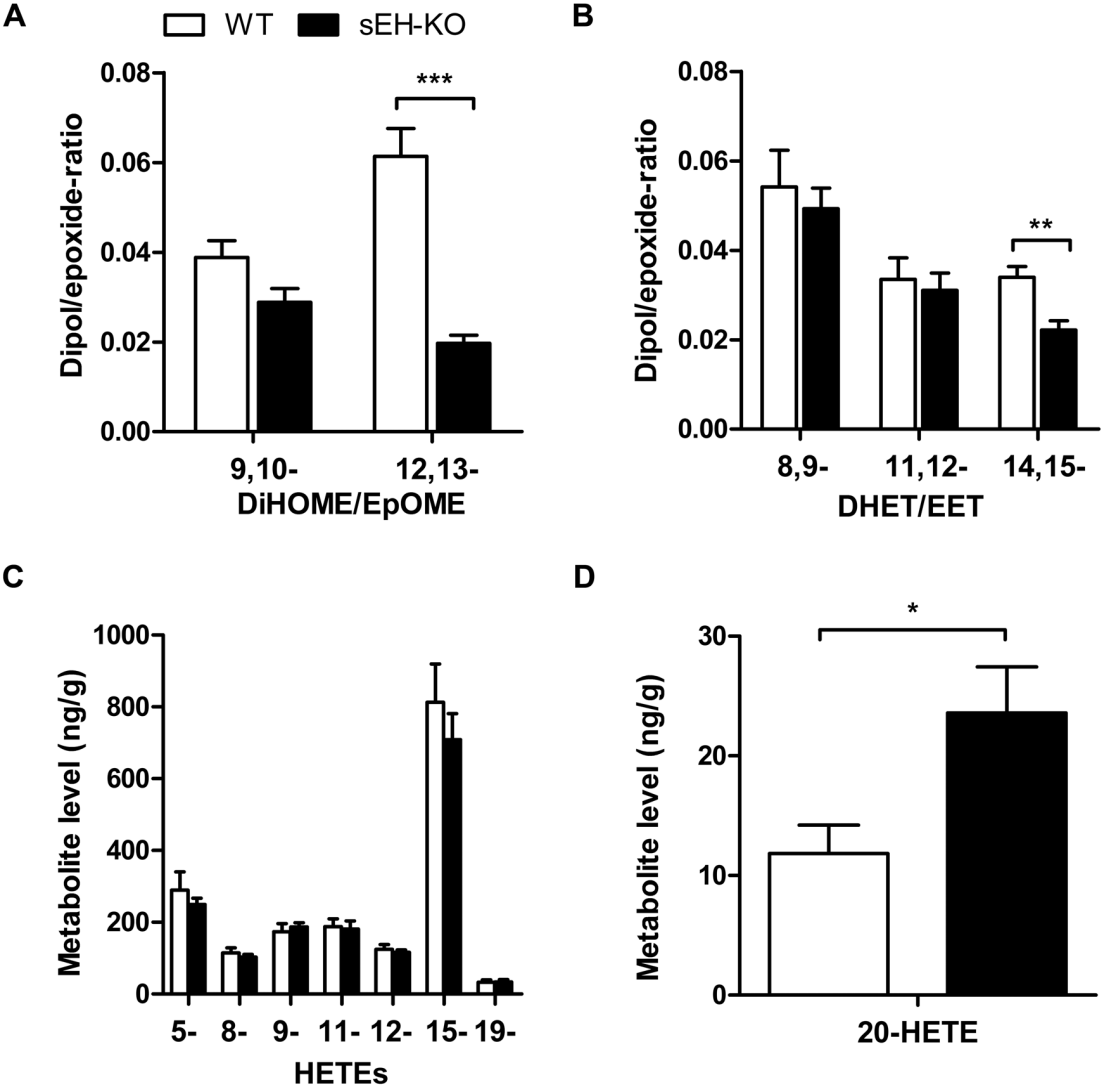
Renal oxylipin profiles in WT and sEH-KO mice. (A): The conversion of linoleic acid-derived epoxides (EpOMEs) to the corresponding vicinal diols (DiHOMEs) was reduced in sEH-KO compared to WT mice as indicated by the decreased DiHOME/EpOME-ratios. (B): The conversion of 14,15-EET to 14,15-DHETs was reduced in sEH-KO compared to WT mice as indicated by the decreased 14,15-DHET/14,15-EET-ratio. (C): The renal levels of AA-derived 5- through 19-HETE were not different in WT and sEH-KO mice. (D): Renal 20-HETE levels were significantly higher in sEH-KO compared with WT mice. Results are given as mean ± SEM (n = 5–7 per group). Statistically significant differences were observed as indicated: * p<0.05, ** p<0.01 vs WT.

**Fig 8:**
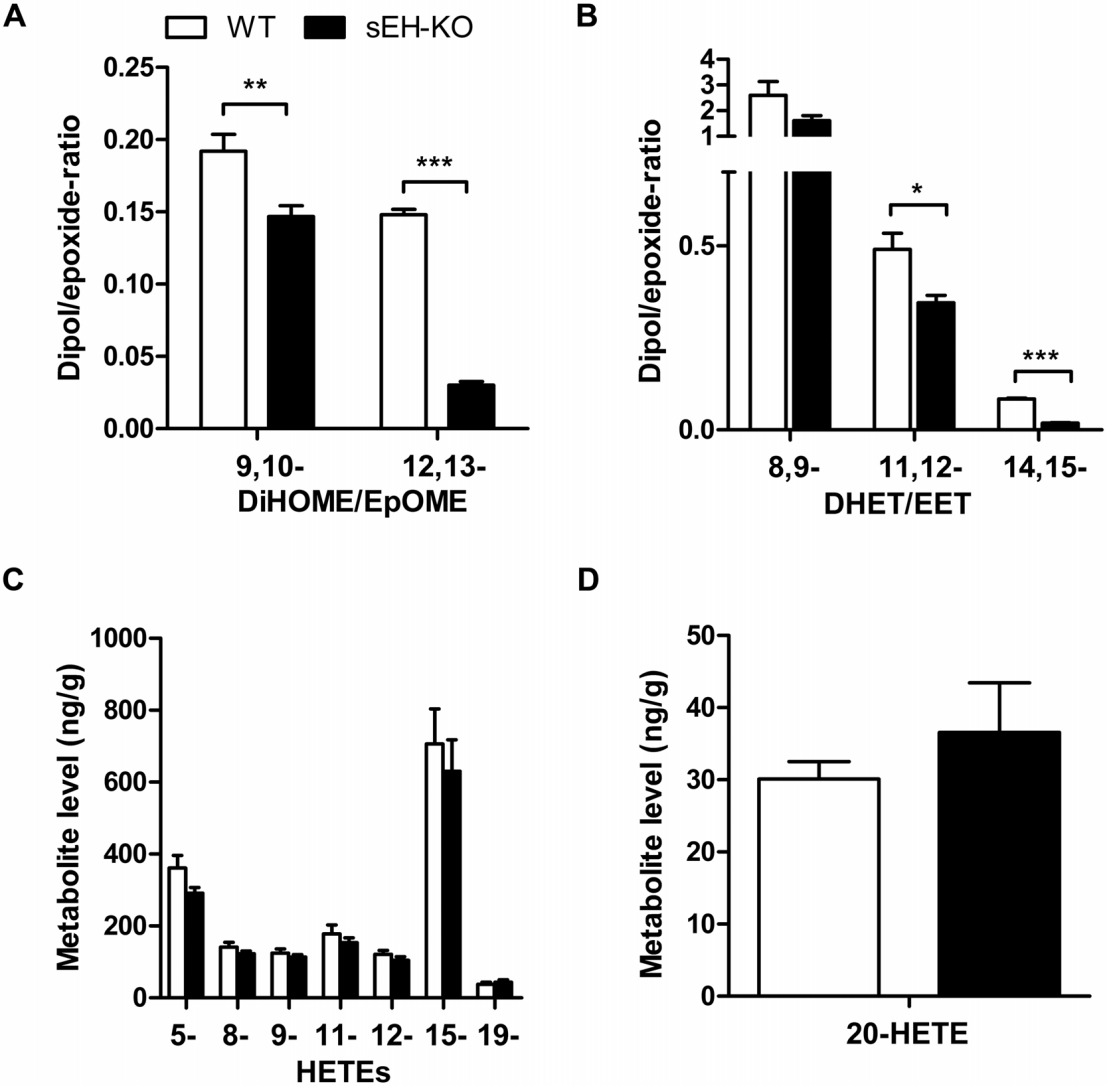
Hepatic oxylipin profiles in WT and sEH-KO mice. (A), (B): Metabolic deficiency of sEH resulted in decreased DiHOME/EpOME- (A) as well as 14,15-DHET/14,15-EET-ratios (B). (C), (D): The hepatic levels of 5- through 19-HETE (C) as well as the hepatic 20-HETE levels (D) were not different in WT and sEH-KO mice. Results are given as mean ± SEM (n = 5–6 per group). Statistically significant differences were observed as indicated: * p<0.05, ** p<0.01 vs WT.

Searching for potential mechanisms predisposing the sEH-KO mice to increased I/R-induced renal damage, we compared the oxylipin profiles of WT and sEH-KO mice under baseline conditions. Our LC-MS/MS analysis included several of the linoleic acid- and AA-derived endogenous substrates and products of sEH-mediated hydrolysis in order to evaluate the direct metabolic consequences of sEH deficiency. Moreover, we determined the levels of various AA-derived monohydroxy-metabolites (HETEs) to gain insight into sEH deficiency-associated alterations in other branches of AA metabolism. The sEH product/substrate-ratios were reduced in sEH-KO compared to WT mice, as congruently demonstrated by the oxylipin profiles of plasma, kidney, and liver samples (Figs 6, 7 and 8; S1, S2 and S3 Tables). In particular, sEH-KO mice displayed markedly increased plasma and tissue levels of the sEH substrates 12,13-epoxyoctadecenoic acid (12,13-EpOME) and 14,15-EET, whereas the corresponding products 12,13-dihydroxyoctadecenoic acid (12,13-DiHOME) and 14,15-DHET were significantly reduced. In contrast to this clear effect of sEH deficiency on the maintenance of epoxygenase metabolites, WT and sEH-KO mice showed almost identical plasma and tissue levels of 5-, 8-, 9-, 11-, 12-, and 15-HETE, indicating that sEH deficiency was not associated with major changes in the formation of monohydroxy-metabolites via 5-, 12- or 15-lipoxygenases and/or AA-autoxidation. Also, the occurrence of 19-HETE, the product of AA (ω-1)-hydroxylation, was not different in WT and sEH-KO mice. Remarkably, however, our data indicate that sEH deficiency was associated with a kidney-specific upregulation of 20-HETE formation. The renal 20-HETE levels were 2-fold higher in sEH-KO than WT mice (Fig 7D and S1 Table). In contrast, plasma 20-HETE levels were decreased and hepatic 20-HETE levels were not significantly different comparing sEH-KO and WT mice (Figs 6D and 8D; S1 and S3 Tables).

### Renal expression of Cyp4a12a was upregulated in sEH-KO mice

**Fig 9:**
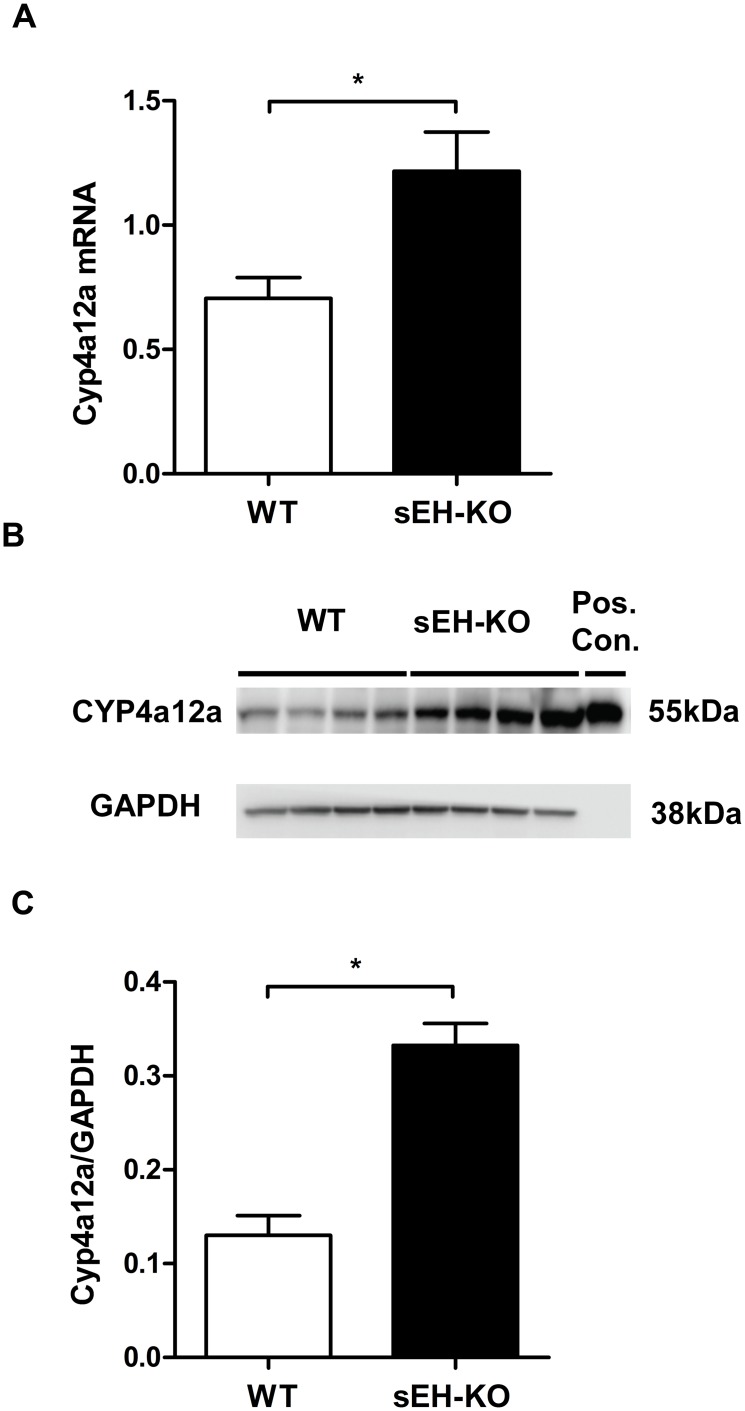
Upregulation of renal Cyp4a12a expression in sEH-KO mice. (A): Quantification of Cyp4a12a mRNA expression in kidneys of WT and sEH-KO mice by TaqMan RT-PCR. (B): A representative Western blot comparing the renal expression of Cyp4a12a protein in WT and sEH-KO mice. (C): Quantification of immunoreactive bands showed 2.5-fold higher Cyp4a12a protein levels in the kidneys of sEH-KO compared to WT mice. Real time-PCR data (A) and Western blot data (C) are given as mean ± SEM (n = 5–7 group). Statistically significant differences were observed as indicated: * p<0.05 vs WT.

Searching for the origin of increased renal 20-HETE levels in sEH-KO mice, we analyzed untreated kidneys from sEH-KO and WT mice for the expression of Cyp4a12a, the major murine 20-HETE generating enzyme [39]. As shown in Fig 9, renal Cyp4a12a expression was about two-fold higher both at the mRNA and protein level in sEH-KO than WT mice. Cyp4a12a mRNA expression levels determined by TaqMan RT-PCR and normalized to GAPDH expression were 0.71±0.08 in WT vs. 1.22±0.15 in sEH-KO mice (*P*<0.05; Fig 9A). The peptide-specific Cyp4a12a antibody recognized a single 55 kDa protein band that co-migrated with recombinant Cyp4a12a in SDS-PAGE (Fig 9B). Quantification of Western blots using GAPDH as loading control showed significantly increased intensities of the Cyp4a12a immunoreactive band in the renal homogenates of sEH-KO compared to WT mice (0.33±0.02 vs 0.13±0.02, *P*<0.05; Fig 9C).

### Localization of Cyp4a12a in the kidney

**Fig 10:**
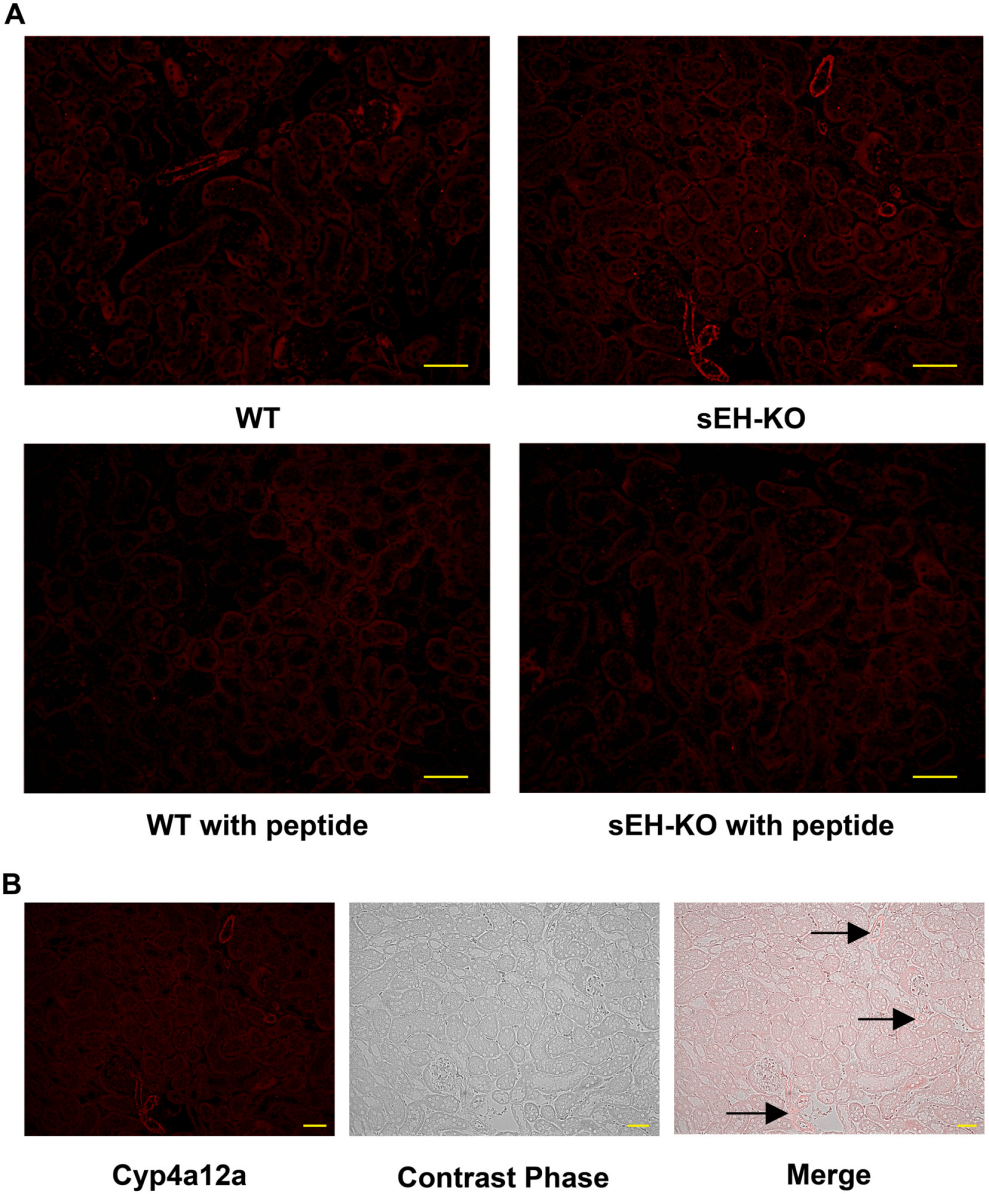
Intrarenal localization of Cyp4a12a protein expression. (A): Representative images of renal sections stained for Cyp4a12a by the immunofluorescence (magnification 200×; scale bar: 50 μm). sEH gene disruption resulted in upregulating the expression of Cyp4a12a in mouse kidneys compared to WT mouse. The signals were blocked by pre-saturating the peptide-specific Cyp4a12a antibody with the corresponding synthetic peptide. (B): Images of a renal section from sEH-KO mice showing how immunostaining relates to the underlying renal structures (magnification 200×; scale bar: 50 μm). Images were taken at the area of renal cortex. Cyp4a12a immunostaining was most intense in the renal vessels (arcuate, interlobar, and interlobular arteries). Faint but specific staining occurred in tubules. No staining was detectable in glomeruli.

To visualize the intrarenal localization of Cyp4a12a, kidney sections were incubated with a peptide-specific Cyp4a12a antibody followed by a fluorescent labeled secondary antibody. Immunostaining occurred in renal vascular and tubular structures and could be blocked at both sites by pre-saturating the Cyp4a12a antibody with the corresponding synthetic peptide (Fig 10A). Tubular immunofluorescence was rather faint and not different comparing WT and sEH-KO mice. The structures displaying clearly enhanced immunostaining in sEH-KO mice represented renal vessels (arcuate, interlobar, and interlobular arteries) as shown in Fig 10B.

## Discussion

In this study, we investigated the effect of global sEH gene disruption on the development of ischemic AKI in mice. Similar to our previous experiments in rat [11], we used a model of unilateral ischemia, i.e. I/R injury was induced after acute uninephrectomy in the remaining kidney. In contrast to our expectation, sEH deficiency did not ameliorate I/R-induced renal damage, but rather aggravated the impairment of kidney function, tubular injury, and inflammatory response. Baseline oxylipin profiling as well as analysis of Cyp4a12a expression revealed increased renal 20-HETE formation as a factor potentially causing the increased susceptibility of sEH-KO mice to I/R-induced renal damage.

In apparent contrast to our findings with the sEH-KO mice, pharmacological inhibition of sEH activity was recently shown to protect against renal I/R injury in mice [33]. Moreover, renoprotective effects of sEH gene deletion were reported in mouse models developing chronic kidney disease [43–45]. There are also several studies indicating that pharmacological sEH inhibition and sEH gene deletion may produce essentially the same beneficial effects as clearly demonstrated in mouse models of myocardial infarction [46,47] and stroke [48,49]. Actually, our study is among the very few indicating that sEH deficiency may also have detrimental effects in certain disease processes. Other examples include reduced survival of sEH-KO mice following cardiac arrest and cardiopulmonary resuscitation [50] and increased albuminuria in mice with progressive renal disease upon pharmacological sEH inhibition [51]. Opposite effects of sEH gene deletion and pharmacological inhibition were observed analyzing angiotensin II-induced cardiac dysfunction and myocardial fibrosis in mice [52]. Resembling the state-of-affairs with renal I/R-injury, cardiac dysfunction and fibrosis were attenuated by sEH inhibition but aggravated by sEH gene deletion [52]. Differences in the effects of sEH gene deletion and pharmacological inhibition were also reported regarding the development of hypoxia-induced pulmonary hypertension [53]. Deletion of the sEH gene eliminates the expression of the whole bi-functional enzyme, whereas the currently developed sEH inhibitors specifically target its C-terminal epoxide hydrolase domain [29]. Accordingly, differences observed comparing the effects of sEH deletion and sEH inhibition may indicate an important role of the N-terminal phosphatase domain in the given disease model as discussed for hypoxia-induced pulmonary hypertension [53]. The function of the phosphatase domain is only partially understood [29]; however, recent findings suggest that the N-terminal domain is involved in regulating the phosphorylation state and activity of endothelial nitric oxide synthase [54,55].

Moreover, both sEH-deletion and sEH-inhibition may cause the development of compensatory mechanisms in response to either increased levels of epoxy-metabolites or reduced levels of the corresponding hydrolysis products. In line with this hypothesis, sEH-inhibition shifted the renal AA metabolism towards the lipoxygenase pathway and failed to elicit renoprotective effects in the 5/6-nephrectomy mouse model [51]. A shift in AA metabolism was also identified as the likely cause for increased angiotensin II-induced myocardial fibrosis in sEH-KO mice compared to pharmacological inhibition of sEH activity in WT mice [52].

EETs function as vasodilators in a variety of vascular beds raising the possibility that systemic hemodynamic effects might impair renal blood flow and thus contribute to the increased renal I/R injury observed in sEH-KO mice. However, like sEH gene deletion also pharmacological sEH inhibition increases endogenous EET levels, but protects against renal I/R injury in mice [33]. Moreover, despite having increased endogenous EET levels, sEH-KO mice display normal blood pressure and show a reduced hypotensive response to LPS challenge [37]. The same study revealed largely increased AA ω-hydroxylase activities in the kidneys of sEH-KO compared to WT mice. Accordingly, it has been suggested that blood pressure homeostasis is achieved in sEH-KO mice by compensatory upregulation of renal 20-HETE formation [37]. Our data confirm and extent these findings. On the one hand, plasma as well as renal and hepatic EpOME/DiHOME and EET/DHET-ratios were higher in sEH-KO than WT mice, thus confirming the expected direct metabolic consequences of sEH deficiency. On the other hand, our LC-MS/MS analysis also showed two-fold higher endogenous 20-HETE levels in the kidneys of sEH-KO compared with WT mice. This indirect “compensatory” effect was obviously kidney-specific considering that the endogenous 20-HETE levels were not significantly higher in the liver and even lower in the plasma of sEH-KO than WT mice. In line with the increased renal 20-HETE content, Cyp4a12a, the major murine 20-HETE generating CYP enzyme, was significantly upregulated both at the mRNA and protein level in the kidneys of sEH-KO compared with WT mice. Importantly, immunohistochemistry indicated renal vessels (arcuate, interlobar, and interlobular arteries) as the major site of increased Cyp4a12a expression in sEH-KO mice.

The mechanisms are currently unclear that lead to the observed upregulation of renal 20-HETE formation in sEH-KO mice. Renal Cyp4a12a expression is largely male-specific and can be further increased by treating C57Bl/6 mice with androgens [39] resulting in increased vascular 20-HETE production [56]. However, male sEH-KO mice feature decreased plasma testosterone levels [57] and the mechanistic link between sEH gene deletion and vascular Cyp4a12a overexpression remains to be elucidated. Interestingly, a very recent study revealed that up-regulation of 20-HETE is a male-specific response, whereas female sEH-KO mice preserve vascular homeostasis by different mechanisms [58].

Taken together, our findings suggest that increased Cyp4a12a-mediated 20-HETE formation in renal vessels might be responsible for the increased susceptibility of male sEH-KO mice to renal I/R-injury. Supporting this notion, vascular overproduction of 20-HETE has the potential of mediating sustained vasoconstriction [21] and to promote inflammatory activation of endothelial cells [59–61]. In line with the proposed detrimental role of 20-HETE in sEH-KO mice, we showed previously that inhibition of 20-HETE formation or action protects against renal I/R injury in uninephrectomized male rats [11]. However, considering the complex vascular and tubular roles of 20-HETE in the kidney and the controversy surrounding its role in renal I/R injury as already described in the introduction part [10–12], we cannot exclude that our findings specifically apply to the model of unilateral ischemia that was used in the present study and also in our previous study with rats [11]. The exact mechanism driving upregulation of renal vascular 20-HETE formation in male sEH-KO mice remains unclear, but is most likely the effect of a compensatory, phenotypic response to the loss of sEH and increased renal EETs. These results further support the notion that finding the delicate balance of modulating EET/HETE in cardiorenal disease remains challenging.

## Supporting Information

S1 Table Comparison of plasma oxylipin profile between WT and sEH-KO mice (ng/ml). (DOCX)

S2 Table Comparison of renal oxylipin profile between WT and sEH-KO mice (ng/g). (DOCX)

S3 Table Comparison of liver oxylipin profile between WT and sEH-KO mice (ng/g). (DOCX)

## References

[1] Bonventre JV, Yang L (2011) Cellular pathophysiology of ischemic acute kidney injury. J Clin Invest 121: 4210–4221. 10.1172/JCI45161 22045571 PMC3204829

[2] Siew ED, Davenport A (2014) The growth of acute kidney injury: a rising tide or just closer attention to detail? Kidney Int. 10.1038/ki.2014.293 PMC4281297 25229340

[3] Karkouti K, Wijeysundera DN, Yau TM, Callum JL, Cheng DC, Crowther M, et al (2009) Acute kidney injury after cardiac surgery: focus on modifiable risk factors. Circulation 119: 495–502. 10.1161/CIRCULATIONAHA.108.786913 19153273

[4] Aydin Z, van Zonneveld AJ, de Fijter JW, Rabelink TJ (2007) New horizons in prevention and treatment of ischaemic injury to kidney transplants. Nephrol Dial Transplant 22: 342–346. 17132706 10.1093/ndt/gfl690

[5] Coca SG, Yusuf B, Shlipak MG, Garg AX, Parikh CR (2009) Long-term risk of mortality and other adverse outcomes after acute kidney injury: a systematic review and meta-analysis. Am J Kidney Dis 53: 961–973. 10.1053/j.ajkd.2008.11.034 19346042 PMC2726041

[6] Hsu CY, Chertow GM, McCulloch CE, Fan D, Ordonez JD, Go AS (2009) Nonrecovery of kidney function and death after acute on chronic renal failure. Clin J Am Soc Nephrol 4: 891–898. 10.2215/CJN.05571008 19406959 PMC2676192

[7] Ishani A, Xue JL, Himmelfarb J, Eggers PW, Kimmel PL, Molitoris BA, et al (2009) Acute kidney injury increases risk of ESRD among elderly. J Am Soc Nephrol 20: 223–228. 10.1681/ASN.2007080837 19020007 PMC2615732

[8] Kusch A, Hoff U, Bubalo G, Zhu Y, Fechner M, Schmidt-Ullrich R, et al (2013) Novel signalling mechanisms and targets in renal ischaemia and reperfusion injury. Acta Physiol (Oxf). 10.1111/apha.12089 23432924

[9] Lameire N, Van Biesen W, Vanholder R (2005) Acute renal failure. Lancet 365: 417–430. 15680458 10.1016/S0140-6736(05)17831-3

[10] Regner KR, Zuk A, Van Why SK, Shames BD, Ryan RP, Falck JR, et al (2009) Protective effect of 20-HETE analogues in experimental renal ischemia reperfusion injury. Kidney Int 75: 511–517. 10.1038/ki.2008.600 19052533 PMC2643317

[11] Hoff U, Lukitsch I, Chaykovska L, Ladwig M, Arnold C, Manthati VL, et al (2011) Inhibition of 20-HETE synthesis and action protects the kidney from ischemia/reperfusion injury. Kidney Int 79: 57–65. 10.1038/ki.2010.377 20962739 PMC3813968

[12] Roman RJ, Akbulut T, Park F, Regner KR (2011) 20-HETE in acute kidney injury. Kidney Int 79: 10–13. 10.1038/ki.2010.396 21157458 PMC3146060

[13] Gross ER, Nithipatikom K, Hsu AK, Peart JN, Falck JR, Campbell WB, et al (2004) Cytochrome P450 omega-hydroxylase inhibition reduces infarct size during reperfusion via the sarcolemmal KATP channel. J Mol Cell Cardiol 37: 1245–1249. 15572055 10.1016/j.yjmcc.2004.10.008

[14] Gross GJ, Falck JR, Gross ER, Isbell M, Moore J, Nithipatikom K (2005) Cytochrome P450 and arachidonic acid metabolites: role in myocardial ischemia/reperfusion injury revisited. Cardiovasc Res 68: 18–25. 15993870 10.1016/j.cardiores.2005.06.007

[15] Miyata N, Seki T, Tanaka Y, Omura T, Taniguchi K, Doi M, et al (2005) Beneficial effects of a new 20-hydroxyeicosatetraenoic acid synthesis inhibitor, TS-011 [N-(3-chloro-4-morpholin-4-yl) phenyl-N'-hydroxyimido formamide], on hemorrhagic and ischemic stroke. J Pharmacol Exp Ther 314: 77–85. 15831442 10.1124/jpet.105.083964

[16] Dunn KM, Renic M, Flasch AK, Harder DR, Falck J, Roman RJ (2008) Elevated production of 20-HETE in the cerebral vasculature contributes to severity of ischemic stroke and oxidative stress in spontaneously hypertensive rats. Am J Physiol Heart Circ Physiol 295: H2455–2465. 10.1152/ajpheart.00512.2008 18952718 PMC2614536

[17] Capdevila JH, Falck JR (2002) Biochemical and molecular properties of the cytochrome P450 arachidonic acid monooxygenases. Prostaglandins Other Lipid Mediat 68–69: 325–344. 12432927 10.1016/s0090-6980(02)00038-2

[18] McGiff JC, Quilley J (1999) 20-HETE and the kidney: resolution of old problems and new beginnings. Am J Physiol 277: R607–623. 10484476 10.1152/ajpregu.1999.277.3.R607

[19] Roman RJ (2002) P-450 metabolites of arachidonic acid in the control of cardiovascular function. Physiol Rev 82: 131–185. 11773611 10.1152/physrev.00021.2001

[20] Konkel A, Schunck WH (2011) Role of cytochrome P450 enzymes in the bioactivation of polyunsaturated fatty acids. Biochim Biophys Acta 1814: 210–222. 10.1016/j.bbapap.2010.09.009 20869469

[21] Imig JD (2013) Epoxyeicosatrienoic acids, 20-hydroxyeicosatetraenoic acid, and renal microvascular function. Prostaglandins Other Lipid Mediat 104–105: 2–7. 10.1016/j.prostaglandins.2013.01.002 23333581 PMC3664103

[22] Wu CC, Gupta T, Garcia V, Ding Y, Schwartzman ML (2014) 20-HETE and blood pressure regulation: clinical implications. Cardiol Rev 22: 1–12. 10.1097/CRD.0b013e3182961659 23584425 PMC4292790

[23] Nilakantan V, Maenpaa C, Jia G, Roman RJ, Park F (2008) 20-HETE-mediated cytotoxicity and apoptosis in ischemic kidney epithelial cells. Am J Physiol Renal Physiol 294: F562–570. 10.1152/ajprenal.00387.2007 18171997 PMC2633439

[24] Fan F, Muroya Y, Roman RJ (2015) Cytochrome P450 eicosanoids in hypertension and renal disease. Curr Opin Nephrol Hypertens 24: 37–46. 10.1097/MNH.0000000000000088 25427230 PMC4260681

[25] Campbell WB, Falck JR (2007) Arachidonic acid metabolites as endothelium-derived hyperpolarizing factors. Hypertension 49: 590–596. 17200437 10.1161/01.HYP.0000255173.50317.fc

[26] Node K, Huo Y, Ruan X, Yang B, Spiecker M, Ley K, et al (1999) Anti-inflammatory properties of cytochrome P450 epoxygenase-derived eicosanoids. Science 285: 1276–1279. 10455056 10.1126/science.285.5431.1276 PMC2720027

[27] Yang B, Graham L, Dikalov S, Mason RP, Falck JR, Liao JK, et al (2001) Overexpression of cytochrome P450 CYP2J2 protects against hypoxia-reoxygenation injury in cultured bovine aortic endothelial cells. Mol Pharmacol 60: 310–320. 11455018 10.1124/mol.60.2.310

[28] Dhanasekaran A, Gruenloh SK, Buonaccorsi JN, Zhang R, Gross GJ, Falck JR, et al (2008) Multiple antiapoptotic targets of the PI3K/Akt survival pathway are activated by epoxyeicosatrienoic acids to protect cardiomyocytes from hypoxia/anoxia. Am J Physiol Heart Circ Physiol 294: H724–735. 18055514 10.1152/ajpheart.00979.2007 PMC2443685

[29] Harris TR, Hammock BD (2013) Soluble epoxide hydrolase: gene structure, expression and deletion. Gene 526: 61–74. 10.1016/j.gene.2013.05.008 23701967 PMC3733540

[30] Imig JD, Hammock BD (2009) Soluble epoxide hydrolase as a therapeutic target for cardiovascular diseases. Nat Rev Drug Discov 8: 794–805. 10.1038/nrd2875 19794443 PMC3021468

[31] Morisseau C, Hammock BD (2013) Impact of soluble epoxide hydrolase and epoxyeicosanoids on human health. Annu Rev Pharmacol Toxicol 53: 37–58. 10.1146/annurev-pharmtox-011112-140244 23020295 PMC3578707

[32] Elmarakby AA (2012) Reno-protective mechanisms of epoxyeicosatrienoic acids in cardiovascular disease. Am J Physiol Regul Integr Comp Physiol 302: R321–330. 10.1152/ajpregu.00606.2011 22116511

[33] Lee JP, Yang SH, Lee HY, Kim B, Cho JY, Paik JH, et al (2012) Soluble epoxide hydrolase activity determines the severity of ischemia-reperfusion injury in kidney. PLoS One 7: e37075 10.1371/journal.pone.0037075 22590647 PMC3349654

[34] Kim J, Yoon SP, Toews ML, Imig JD, Hwang SH, Hammock BD, et al (2015) Pharmacological inhibition of soluble epoxide hydrolase prevents renal interstitial fibrogenesis in obstructive nephropathy. Am J Physiol Renal Physiol 308: F131–139. 10.1152/ajprenal.00531.2014 25377915 PMC4338924

[35] Lee SH, Lee J, Cha R, Park MH, Ha JW, Kim S, et al (2008) Genetic variations in soluble epoxide hydrolase and graft function in kidney transplantation. Transplant Proc 40: 1353–1356. 10.1016/j.transproceed.2008.03.137 18589104

[36] Lee JP, Yang SH, Kim DK, Lee H, Kim B, Cho JY, et al (2011) In vivo activity of epoxide hydrolase according to sequence variation affects the progression of human IgA nephropathy. Am J Physiol Renal Physiol 300: F1283–1290. 10.1152/ajprenal.00733.2010 21429967

[37] Luria A, Weldon SM, Kabcenell AK, Ingraham RH, Matera D, Jiang H, et al (2007) Compensatory mechanism for homeostatic blood pressure regulation in Ephx2 gene-disrupted mice. J Biol Chem 282: 2891–2898. 17135253 10.1074/jbc.M608057200 PMC2040337

[38] Monti J, Fischer J, Paskas S, Heinig M, Schulz H, Gosele C, et al (2008) Soluble epoxide hydrolase is a susceptibility factor for heart failure in a rat model of human disease. Nat Genet 40: 529–537. 10.1038/ng.129 18443590 PMC7370537

[39] Muller DN, Schmidt C, Barbosa-Sicard E, Wellner M, Gross V, Hercule H, et al (2007) Mouse Cyp4a isoforms: enzymatic properties, gender- and strain-specific expression, and role in renal 20-hydroxyeicosatetraenoic acid formation. Biochem J 403: 109–118. 17112342 10.1042/BJ20061328 PMC1828894

[40] Wei Q, Dong Z (2012) Mouse model of ischemic acute kidney injury: technical notes and tricks. Am J Physiol Renal Physiol 303: F1487–1494. 10.1152/ajprenal.00352.2012 22993069 PMC3532486

[41] Arnold C, Markovic M, Blossey K, Wallukat G, Fischer R, Dechend R, et al (2010) Arachidonic Acid-Metabolizing Cytochrome P450 Enzymes Are Targets of Omega-3 Fatty Acids. J Biol Chem 285: 32720–31733. 10.1074/jbc.M110.118406 20732876 PMC2963419

[42] Fischer R, Konkel A, Mehling H, Blossey K, Gapelyuk A, Wessel N, et al (2014) Dietary omega-3 fatty acids modulate the eicosanoid profile in man primarily via the CYP-epoxygenase pathway. J Lipid Res 55: 1150–1164. 24634501 10.1194/jlr.M047357 PMC4031946

[43] Manhiani M, Quigley JE, Knight SF, Tasoobshirazi S, Moore T, Brands MW, et al (2009) Soluble epoxide hydrolase gene deletion attenuates renal injury and inflammation with DOCA-salt hypertension. Am J Physiol Renal Physiol 297: F740–748. 10.1152/ajprenal.00098.2009 19553349 PMC2739707

[44] Elmarakby AA, Faulkner J, Al-Shabrawey M, Wang MH, Maddipati KR, Imig JD (2011) Deletion of soluble epoxide hydrolase gene improves renal endothelial function and reduces renal inflammation and injury in streptozotocin-induced type 1 diabetes. Am J Physiol Regul Integr Comp Physiol 301: R1307–1317. 10.1152/ajpregu.00759.2010 21832210 PMC3213948

[45] Kim J, Imig JD, Yang J, Hammock BD, Padanilam BJ (2014) Inhibition of soluble epoxide hydrolase prevents renal interstitial fibrosis and inflammation. Am J Physiol Renal Physiol 307: F971–980. 10.1152/ajprenal.00256.2014 25164080 PMC4200297

[46] Motoki A, Merkel MJ, Packwood WH, Cao Z, Liu L, Iliff J, et al (2008) Soluble epoxide hydrolase inhibition and gene deletion are protective against myocardial ischemia-reperfusion injury in vivo. Am J Physiol Heart Circ Physiol 295: H2128–2134. 10.1152/ajpheart.00428.2008 18835921 PMC2614571

[47] Batchu SN, Lee SB, Samokhvalov V, Chaudhary KR, El-Sikhry H, Weldon SM, et al (2012) Novel soluble epoxide hydrolase inhibitor protects mitochondrial function following stress. Can J Physiol Pharmacol 90: 811–823. 10.1139/y2012-082 22624559

[48] Zhang W, Koerner IP, Noppens R, Grafe M, Tsai HJ, Morisseau C, et al (2007) Soluble epoxide hydrolase: a novel therapeutic target in stroke. J Cereb Blood Flow Metab 27: 1931–1940. 17440491 10.1038/sj.jcbfm.9600494 PMC2664093

[49] Zhang W, Otsuka T, Sugo N, Ardeshiri A, Alhadid YK, Iliff JJ, et al (2008) Soluble epoxide hydrolase gene deletion is protective against experimental cerebral ischemia. Stroke 39: 2073–2078. 10.1161/STROKEAHA.107.508325 18369166 PMC2654189

[50] Hutchens MP, Nakano T, Dunlap J, Traystman RJ, Hurn PD, Alkayed NJ (2008) Soluble epoxide hydrolase gene deletion reduces survival after cardiac arrest and cardiopulmonary resuscitation. Resuscitation 76: 89–94. 17728042 10.1016/j.resuscitation.2007.06.031 PMC2585367

[51] Jung O, Jansen F, Mieth A, Barbosa-Sicard E, Pliquett RU, Babelova A, et al (2010) Inhibition of the soluble epoxide hydrolase promotes albuminuria in mice with progressive renal disease. PLoS One 5: e11979 10.1371/journal.pone.0011979 20694143 PMC2915917

[52] Li L, Li N, Pang W, Zhang X, Hammock BD, Ai D, et al (2014) Opposite effects of gene deficiency and pharmacological inhibition of soluble epoxide hydrolase on cardiac fibrosis. PLoS One 9: e94092 10.1371/journal.pone.0094092 24718617 PMC3981766

[53] Keseru B, Barbosa-Sicard E, Schermuly RT, Tanaka H, Hammock BD, Weissmann N, et al (2010) Hypoxia-induced pulmonary hypertension: comparison of soluble epoxide hydrolase deletion vs. inhibition. Cardiovasc Res 85: 232–240. 10.1093/cvr/cvp281 19679679 PMC2860707

[54] Hou HH, Hammock BD, Su KH, Morisseau C, Kou YR, Imaoka S, et al (2012) N-terminal domain of soluble epoxide hydrolase negatively regulates the VEGF-mediated activation of endothelial nitric oxide synthase. Cardiovasc Res 93: 120–129. 10.1093/cvr/cvr267 22072631 PMC3243038

[55] Hou HH, Liao YJ, Hsiao SH, Shyue SK, Lee TS (2015) Role of phosphatase activity of soluble epoxide hydrolase in regulating simvastatin-activated endothelial nitric oxide synthase. Sci Rep 5: 13524 10.1038/srep13524 26304753 PMC4548251

[56] Wu CC, Mei S, Cheng J, Ding Y, Weidenhammer A, Garcia V, et al (2013) Androgen-Sensitive Hypertension Associates with Upregulated Vascular CYP4A12-20-HETE Synthase. J Am Soc Nephrol. 10.1681/ASN.2012070714 PMC3736709 23641057

[57] Luria A, Morisseau C, Tsai HJ, Yang J, Inceoglu B, De Taeye B, et al (2009) Alteration in plasma testosterone levels in male mice lacking soluble epoxide hydrolase. Am J Physiol Endocrinol Metab 297: E375–383. 10.1152/ajpendo.00131.2009 19458064 PMC2724109

[58] Vanella L, Canestraro M, Lee CR, Cao J, Zeldin DC, Schwartzman ML, et al (2015) Soluble epoxide hydrolase null mice exhibit female and male differences in regulation of vascular homeostasis. Prostaglandins Other Lipid Mediat 120: 139–147. 10.1016/j.prostaglandins.2015.04.004 25908301 PMC4575626

[59] Cheng J, Wu CC, Gotlinger KH, Zhang F, Falck JR, Narsimhaswamy D, et al (2010) 20-hydroxy-5,8,11,14-eicosatetraenoic acid mediates endothelial dysfunction via IkappaB kinase-dependent endothelial nitric-oxide synthase uncoupling. J Pharmacol Exp Ther 332: 57–65. 10.1124/jpet.109.159863 19841472 PMC2802478

[60] Ishizuka T, Cheng J, Singh H, Vitto MD, Manthati VL, Falck JR, et al (2008) 20-Hydroxyeicosatetraenoic acid stimulates nuclear factor-kappaB activation and the production of inflammatory cytokines in human endothelial cells. J Pharmacol Exp Ther 324: 103–110. 17947496 10.1124/jpet.107.130336

[61] Inoue K, Sodhi K, Puri N, Gotlinger KH, Cao J, Rezzani R, et al (2009) Endothelial-specific CYP4A2 overexpression leads to renal injury and hypertension via increased production of 20-HETE. Am J Physiol Renal Physiol 297: F875–884. 10.1152/ajprenal.00364.2009 19675180 PMC2775578

