# Supplementary material for: Renal Ischemia/Reperfusion Injury in Soluble Epoxide Hydrolase-Deficient Mice
Source: PLoS One. 2016 Jan 4;11(1):e0145645. doi: 10.1371/journal.pone.0145645 (PMC4699807; doi:10.1371/journal.pone.0145645)
Supplement: S1 Table — (DOCX) [file pone.0145645.s001.docx]

**S1 Table: Comparison of plasma oxylipin profile between WT and sEH-KO mice (ng/ml)**

|  | **WT** | **sEH-KO** |
| --- | --- | --- |
| **Epoxygenase metabolism** | | |
| 12,13-EpOME | 31.39±8.12 | 131.6±16.94*** |
| 9,10-EpOME | 20.57±4.82 | 40.30±7.32 |
| 14,15-EET | 4.00± 0.55 | 9.39±0.29*** |
| 11,12-EET | 1.35±0.21 | 1.73±0.18 |
| 8,9-EET | 1.97±0.25 | 3.43±0.17*** |
| 5,6-EET | 0.21±0.13 | 0.20±0.07 |
| **Soluble epoxide hydrolase metabolism** | | |
| 12,13-DiHOME | 31.63±9.60 | 12.59±4.10 |
| 9,10-DiHOME | 8.99±2.59 | 4.71±1.01 |
| 14,15-DHET | 0.68±0.07 | 0.47±0.07 |
| 11,12-DHET | 0.54±0.04 | 0.42±0.04 |
| 8,9-DHET | 1.35±0.11 | 1.19±0.09 |
| 5,6-DHET | 3.34±0.34 | 4.42±0.32* |
| **ω/( ω1)-Hydroxylase metabolism** | | |
| 20-HETE | 1.50±0.13 | 1.05±0.11* |
| 19-HETE | 8.49±2.24 | 8.50±1.45 |
| **Other monohydroxy metabolites** | | |
| 15-HETE | 28.64±3.85 | 31.12±3.03 |
| 12-HETE | 19.14±6.70 | 16.75±1.92 |
| 11-HETE | 7.92±1.08 | 10.07±0.94 |
| 9-HETE | 7.25±0.82 | 9.53±1.05 |
| 8-HETE | 8.06±0.95 | 10.18±1.26 |
| 5-HETE | 22.68±2.12 | 27.93±2.42 |

EET, epoxyeicosatrienoic acid; DHET, dihydroxyeicosatrienoic acid; HETE, hydroxyeicosatetraenoic acid; EpOME, epoxyoctadecenoic acid; DiHOME, dihydroxyoctadecenoic acid. Data are given as mean ± SEM (n=5-6 per group). * p<0.05, ** p<0.01
