# Supplementary material for: Renal Ischemia/Reperfusion Injury in Soluble Epoxide Hydrolase-Deficient Mice
Source: PLoS One. 2016 Jan 4;11(1):e0145645. doi: 10.1371/journal.pone.0145645 (PMC4699807; doi:10.1371/journal.pone.0145645)
Supplement: S2 Table — (DOCX) [file pone.0145645.s002.docx]

**S2 Table: Comparison of renal oxylipin profile between WT and sEH-KO mice (ng/g)**

|  | **WT** | **sEH-KO** |
| --- | --- | --- |
| **Epoxygenase metabolism** | | |
| 12,13-EpOME | 247.1±25.14 | 525.4±58.59** |
| 9,10-EpOME | 214.2±25.64 | 300.9±26.37* |
| 14,15-EET | 345.4± 38.27 | 361.8±17.25 |
| 11,12-EET | 152.3±18.82 | 154.1±10.81 |
| 8,9-EET | 134.7±14.36 | 127.6±9.26 |
| 5,6-EET | 141.3±33.19 | 123.6±11.62 |
| **Soluble epoxide hydrolase metabolism** | | |
| 12,13-DiHOME | 14.87±1.55 | 10.08±1.09* |
| 9,10-DiHOME | 8.24±1.05 | 8.57±1.03 |
| 14,15-DHET | 11.49±0.87 | 8.20±1.01* |
| 11,12-DHET | 4.76±0.38 | 4.74±0.61 |
| 8,9-DHET | 7.01±0.73 | 6.30±0.73 |
| 5,6-DHET | 44.90±3.30 | 46.17±5.48 |
| **ω/( ω1)-Hydroxylase metabolism** | | |
| 20-HETE | 11.83±2.38 | 23.59±3.85* |
| 19-HETE | 32.79±6.58 | 33.71±6.88 |
| **Other monohydroxy metabolites** | | |
| 15-HETE | 812.5±106.9 | 709.1±72.32 |
| 12-HETE | 124.3±13.52 | 116.2±6.97 |
| 11-HETE | 187.7±22.09 | 181.4±22.15 |
| 9-HETE | 173.6±22.28 | 187.3±11.36 |
| 8-HETE | 114.7±14.47 | 102.8±7.21 |
| 5-HETE | 289.7±50.61 | 249.8±17.70 |

EET, epoxyeicosatrienoic acid; DHET, dihydroxyeicosatrienoic acid; HETE, hydroxyeicosatetraenoic acid; EpOME, epoxyoctadecenoic acid; DiHOME, dihydroxyoctadecenoic acid. Data are given as mean ± SEM (n=5-7 per group). * p<0.05, ** p<0.01
