# Supplementary material for: Renal Ischemia/Reperfusion Injury in Soluble Epoxide Hydrolase-Deficient Mice
Source: PLoS One. 2016 Jan 4;11(1):e0145645. doi: 10.1371/journal.pone.0145645 (PMC4699807; doi:10.1371/journal.pone.0145645)
Supplement: S3 Table — (DOCX) [file pone.0145645.s003.docx]

**S3 Table: Comparison of liver oxylipin profile between WT and sEH-KO mice (ng/g)**

|  | **WT** | **sEH-KO** |
| --- | --- | --- |
| **Epoxygenase metabolism** | | |
| 12,13-EpOME | 343.5±19.72 | 1742±198.4*** |
| 9,10-EpOME | 144.7±8.38 | 259.0±26.64** |
| 14,15-EET | 231.3± 17.82 | 428.2±24.36*** |
| 11,12-EET | 41.83±4.77 | 53.60±6.08 |
| 8,9-EET | 21.81±5.59 | 28.92±6.78 |
| 5,6-EET | 8.19±1.41 | 17.82±3.24* |
| **Soluble epoxide hydrolase metabolism** | | |
| 12,13-DiHOME | 50.53±3.21 | 52.96±4.66 |
| 9,10-DiHOME | 27.54±1.66 | 37.65±4.07 |
| 14,15-DHET | 19.68±1.72 | 8.91±0.75*** |
| 11,12-DHET | 19.79±1.39 | 18.19±1.47 |
| 8,9-DHET | 46.20±2.37 | 40.52±2.66 |
| 5,6-DHET | 45.99±5.30 | 56.24±3.16 |
| **ω/( ω1)-Hydroxylase metabolism** | | |
| 20-HETE | 30.09±2.42 | 36.57±6.85 |
| 19-HETE | 37.70±6.21 | 43.96±6.63 |
| **Other monohydroxy metabolites** | | |
| 15-HETE | 706.3±97.56 | 630.7±87.48 |
| 12-HETE | 121.0±10.76 | 104.8±9.46 |
| 11-HETE | 178.1±25.03 | 153.8±13.59 |
| 9-HETE | 124.9±11.47 | 114.1±6.20 |
| 8-HETE | 141.3±13.15 | 123.1±6.97 |
| 5-HETE | 360.9±35.28 | 291.0±15.94 |
|  |  |  |

EET, epoxyeicosatrienoic acid; DHET, dihydroxyeicosatrienoic acid; HETE, hydroxyeicosatetraenoic acid; EpOME, epoxyoctadecenoic acid; DiHOME, dihydroxyoctadecenoic acid. Data are given as mean ± SEM (n=5-6 per group). * p<0.05, ** p<0.01
